# Factors influencing the well-being of Central and Eastern European university teachers: the role of physical activity and the sources of stress and resources in the workplace

**DOI:** 10.1186/s12889-025-24241-7

**Published:** 2025-09-01

**Authors:** Klára Kovács, Szilvia Borbély, Beáta Dobay, Szabolcs Halasi, Ildikó Vajda, Gabriella Hideg

**Affiliations:** 1 MTA-DE-Parent-Teacher Cooperation Research Group, Institute of Education and Cultural Management, Faculty of Humanities, University of Debrecen, Egyetem sqr. 1, Debrecen, 4032 Hungary; 2 Institute of Physical Education, University of Nyíregyháza, Nyíregyháza, Hungary; 3 Department of Kindergarten and Teacher Training, J. Selye University, Komarno, Slovakia; 4 Institute of Arts and Sports Sciences, Benedek Elek Faculty of Pedagogy, University of Sopron, Sopron, Hungary; 5 Hungarian Language Teacher Training Faculty, University of Novi Sad, Subotica, Serbia; 6 Faculty of Pedagogy, Gál Ferenc University, Szarvas, Hungary; 7 Faculty of Health Sciences, University of Pécs, Pécs, Hungary

**Keywords:** Wellbeing, Physical activity (PA), Stress and resources in the workplace, University teachers, Central and Eastern Europe (CEE)

## Abstract

**Background:**

While previously a university education career seemed like a predictable, relatively stress-free, flexible, socially recognized profession, this today is no longer the case. Where it once was a job which protected its teachers from all sorts of workplace sources of stress like uncertainty, low work control, it now no longer offers such shielding.

**Methods:**

In our study, based on the Jobs-Demands and Resources Theory, we examined the backgrounds and predictor roles of institutional stress sources and resources, and physical activity as an individual asset, in the wellbeing of teachers. For our analyses, we used the CEETHE 2023 research database. Within its framework and with the help of an online survey, we mapped the teacher work characteristics of Hungarian, Slovakian, Ukrainian, Romanian, and Serbian higher education institution teachers (*N* = 821).

**Result:**

According to our results, emotional exhaustion is the most critical negative predictor, while work engagement is the most important positive predictor of teachers wellbeing. In an indirect way, physical activity contributes to wellbeing by decreasing certain sources of stress.

**Conclusions:**

If the university seek to improve its employees’ wellbeing, it would be equally important to emphasize institutional management support. This support would provide clear-cut expectations and tasks, transparent institutional strategies, goals, and plans.

## Background

The health, wellbeing, and satisfaction of teachers, as employees of higher education institutions, are important not only on an individual level, but also on an institutional level. For, they help a university to function properly [5]. Thus, it is important to discover what work-related and personal stress-sources and resources affect teacher wellbeing. In our study, our aim is answering this niche question by examining Central and Eastern European (Hungarian, Slovakian, Ukrainian, Romanian, and Serbian) teachers. In order to come up with research questions, the Job demands and resources theory (JD-R theory) was used. The JD-R theory is a theoretical framework that helps to explain and understand the relationship between workplace characteristics, employee performance, and wellbeing [4]. Work resources and requirements are also scrutinized. Work resources contribute to the personal development and learning of the employee as intrinsic motivation, and to achieving goals at an institutional level as extrinsic motivation. Work requirements are inversely related to commitment to work. A high level of work requirements can lead to chronic stress, fatigue, and emotional exhaustion, which can reduce commitment to work, and through this, job satisfaction [19]. JD-R theory examines two parallel processes surrounding the workplace. One process puts the health at risk, with work requirements not only exhausting employee physical and psychological resources, thus negatively affecting their wellbeing and performance, but also causing emotional exhaustion and burnout. The other deals with coping strategies stored up as personal resources which can help employees cope with the stress caused by the workplace.

Research on the subjective wellbeing of university teachers has focused primarily on the relationship between job demands and resources. The interaction between job demands and resources is critical in shaping motivational processes that lead to job engagement and wellbeing, while reducing job stress leading to burnout [36]. In our study, we identify as job demands the various sources of workplace stress (workload, performance evaluation, and diversity of teacher roles, work–personal life conflicts, and the emotional exhaustion at work). Meanwhile, institutional support and engagement in work are considered workplace resources and level of physical activity is a personal resource.

The concept of wellbeing in our research is related to Ed Diener’s idea of hedonic subjective wellbeing. According to his definition, subjective wellbeing refers to people’s evaluation of their own lives, the result of feeling in control of one’s own life, of being able to pursue one’s own goals, of being able to engage in activities and social relationships that one finds interesting and enjoyable [14]. Diener’s notion of subjective wellbeing has a cognitive and an affective domain. The cognitive component refers to an individual’s evaluation of various aspects of his or her life. The affective component focuses on emotions (positive and negative), their experiences, affections and moods. Positive emotions depict wellbeing, a balance between positive and negative emotions which contributes to satisfaction with life. Closely related is Park et al.‘s [38] definition of emotional wellbeing, which is a multi-dimensional composite that encompasses how positive an individual feels in general and about life overall. In our research, we used this definition to ask respondents about the frequency with which university teachers had experienced different positive and negative emotions in the past few weeks.

### The role of physical activity and workplace stress sources in employee wellbeing

Appearing with ever-increasing frequency in the focus of international academic researchers is the testing of workplace satisfaction and commitment. Recognition of the work specifically improves satisfaction [40]. According to Dallmeyer et al., weekly leisure-time physical activity (LTPA) has a positive effect on work satisfaction, also influencing health, while it is also heavily impacted by workplace stress [12].

Working longer and harder, however, can be harmful to employees’ health and wellbeing. The pressure of time led to fatigue on a weekly basis. This fatigue was found to be mediated by extending work, but not by intensifying work. Interestingly, teachers with high levels of dispositional perfectionistic concerns and strivings were more likely to engage in working harder each week. Notably, dispositional perfectionism did not affect how teachers responded to the pressure of time [22].

Based on Cohen & Wills’ [9] stress-buffering hypothesis many researchers confirm that regular physical activity has a positive effect on an individual in overcoming stress, promoting mental health, and bettering someone’s ability to work [8, 17, 18, 25, 41]. The essence of the stress-buffering hypothesis is that social support can alleviate the negative effects of stress, particularly in situations where the individual is exposed to intense strain. Social support functions as a ‘buffer’ that mitigates the negative physiological and psychological consequences triggered by stress [9]. Salmon’s [41] research extended the context of the stress-buffering hypothesis by demonstrating that physical activity—similarly to social support—can reduce these negative effects, thereby providing a more effective coping strategy. According to Salmon [41], regular physical exercise activates biochemical processes that improve mood and reduce anxiety. The release of endorphins and other neurotransmitters can thus lessen the intensity of stress responses. Furthermore, physical activity may help regulate the levels of stress hormones such as cortisol. The findings also highlighted that regular exercise promotes the body’s adaptive response to stress, which increases resilience to stress in the long run. This means that not only is the immediate stress reflex improved, but everyday management also becomes more effective. The stress-reducing mechanisms of physical activity include regulating the body’s stress responses, such as lowering the activity of the sympathetic nervous system. In this way, exercise can help to assuage the physiological reactions to stress, contributing to an individual’s overall well-being. When compared to Cohen & Wills’ [9] theory, while social support provides a protective effect in a psychological and emotional sense, physical activity exerts its impact directly through physiological processes. Both factors—though operating in different ways—reduce the harmful effects of stress. Thus, both, in an integrated way, add to overall well-being and effective stress management.

Moreover, regular physical activity influences anxiety-related disorders and stress in a good way. Because of the broad applicability thereof, it can be considered a transdiagnostic approach to be effectively employed in the treatment of the aforementioned various anxiety disorders. In addition to health status, physical activity has a significant positive impact on an individual’s overall wellbeing and function [13, 26, 32]. In this context, it reduces the risk of cardiovascular disease and premature death and contributes to the development and maintenance of a healthy lifestyle [24]. The practice of sport and physical activities and leisure was proved to be a protective factor against burnout syndrome among Cameroon teachers [33].

Contrastingly, Cortés-Denia et al. [10] and Chekroud et al. [7] found such complex tests, which simultaneously measure a worker’s physical activity, workplace work output, the effects of stress on mental health, and their mutual effects upon one another. All in all, it was confirmed that physical activity and workplace stress have a positive relationship on the organizational system subscale. This refers to the organization’s operational system, the conflicts and the rational communication that occur within that system.

All things considered, the conclusion we can come to, based on the results, is that workplace stress can extinguish advantages of regular physical exercise. What is more, the employee’s vigor at work decreases with the presence of stress and the lack of physical activity.

Due to the inconsistency of these results in our study, we first examined the forms of physical activity using an International Physical Activity Questionnaire (IPAQ) [11]. Saraiva et al. [42] checked the physical activity of Brazilian teachers from a demographics and work-related background aspect. According to their results, female teachers, professors, teachers with doctorates, and employees in the field of humanities were less active. Through the JD-R theory, along with various stress sources, their ties to the indicators of wellbeing have also been checked in multiple other countries like, Britain and Australia [5, 16, 27] Pakistan [3], Saudi Arabia [2], China [19] and Nigeria [23]. In our study, we examined in a model not only the variables from these researches, but also we included physical activity as a personal resource. There are certain studies too in which teachers examined the background of physical activity [23, 28, 31, 42], in these, however, we can rarely find results regarding the connection between wellbeing, workplace stress sources and resources.

From multiple angles, including this one, our study can be seen as novel. For, in contrast to the student population, few studies deal with the physical activity of academics and the problem of their inactivity [31].

The geographical area (CEE) can be considered a novelty as well, as it is a region that contains several countries that are both similar and different. They are like one another having similar cultural roots, historical antecedents, and similar social-economic situations. Yet, they are estranged from each other in their many peculiarities. The societies in these countries are still struggling with the challenges of political-economic transformation, having been affected by the economic recession, COVID-19, wars, and other such events. In CEE countries, in particular, cardiovascular disease mortality is the highest in the world [34], cancer is the leading cause of death according to GLOBOCAN 2020 [15], and health condition of these post-socialist countries lags behind the European Union average [6]. These all come as the results of the socio-economic challenges, social inequalities, the inactive and unhealthy lifestyles, and the differences in health systems in these nations.

As a result of the 1920 Treaty of Trianon, the cross-border regions of Hungary were partitioned off to the neighbouring countries (now the Highlands in Slovakia, Transcarpathia in Ukraine, Vojvodina in Serbia, Transylvania and Partium in Romania), which is why these regions have a large number of Hungarian minorities and they have their own higher education institutions [39]. There are also majority higher education institutions in these regions where majority and minority students study, and teachers work together. Thus, the geographical area under study is a special, multicultural region where ethnic, cultural and economic differences influence the work of higher education institutions and university teachers. Previously, however, there was no research done about the working conditions, job-demands and resources, and the influencing factors of wellbeing among university teachers in this region. Though similar research has been conducted among young researchers in Hungary [37], it is not the same population as the teachers.

Our aim is to discover the background of the physical activity of teachers working in the examined countries, and to uncover their connections to workplace sources of stress and resources, as well as to wellbeing. In our study, the questions are:

1. What differences are there among the teacher groups that were formed along physical activity, regarding demographics and work characteristics?
2. What differences can be found in workplace stress-sources and resources, in work engagement, in exhaustion and wellbeing, among the teacher groups characterized by various levels of physical activity?
3. What connections exist between physical activity, stress sources and resources, emotional exhaustion, work engagement, along with wellbeing?

Based on the previous research results in the literature, we assume that moderate or high PA, workplace resources, and work engagement will be positive predictors of wellbeing, while stress sources and exhaustion in work will turn out to be negative predictors. PA will reduce the stress sources and exhaustion and contribute to work engagement and workplace resources. It is also assumed that there will be significant differences in PA by the most important demographic factors and work characteristics. The following hypotheses were formulated:

1. Based on Saraiva et al. [42] results, younger male teachers without an academic degree and lower position will be more active physically.
2. Based on the Cohen & Wills’ [9] stress-buffering hypothesis and previous research results on the role of PA [8, 12, 17, 18, 25, 41] the level of workplace stress sources and emotional exhaustion decreases, while perception of workplace resources, level of engagement increase with the level of PA.
3. Based on the JD-R theory [4] and the results of Sahibzada & Khawrin [40] workplace stressors and exhaustion in work decrease, while PA, workplace resources and work engagement increase the level of teachers’ wellbeing.

## Method

### Data collection and sample

Our detailed questionnaire survey examined the factors that characterize academics’ teaching and research work, working conditions, workplace outcomes, health, lifestyle, institutional, family, and social backgrounds (over 300 items). The questionnaire being translated into Serbian, Slovakian, Ukrainian and Romanian, and being sent, with the permission of the head of the institution, 2 or 3 times via the university’s online learning system to all teachers of the institutions surveyed in the spring of 2023, ensured a probability sample (all teachers had a chance to be included in the sample).

We examined the higher education establishments of two disadvantaged regions in Hungary, the Northern Great Plains Region (the University of Debrecen, the Debrecen Reformed Theological University, and the University of Nyíregyháza), and the Southern Transdanubia Region (the University of Pécs). When examining cross-border institutions, we targeted minority Hungarian institutions. The examined universities were as follows: the University of Novi Sad in Serbia, the J. Selye University and the University of Presov and the University of Trnava in Slovakia, the Partium Christian University, the Oradea State University, the Emanuel University, the Sapienta Hungarian University of Transylvania, and the Babeş-Bolyai University and their satellite branches in Romania, the Ferenc Rákoczi II Transcarpathian Hungarian College and Uzhhorod National University in Ukraine. Though we examined Hungarian minority institutions, we still sent out the survey in the language of the majority. Teachers from these regions filled out the survey, even those working in institutions from inner Ukraine completed the survey (*n* = 62). Regarding teachers in Ukraine, most of the respondents in the Ukrainian subsample live and work in the Transcarpathian region, though several teachers from the other regions of Ukraine moved because of the war. If they were excluded from the sample, the Ukrainian sub-sample would be reduced by half. Based on their answers, we could collect a lot of valuable information about the special situation of university teachers’ work during the war. It has a significant impact on their working conditions and wellbeing, which is the most important question in our research. We therefore decided to keep the respondents from inside Ukraine for further analysis.

To reach representativity we strove for a 10% sample for each institution devoid of faculties, and for each faculty in larger universities. We reached much larger samples with the smaller universities and colleges, where fewer than 100 were employed. In the interest of reaching probability sample, where we had opportunity and permission, we sent the survey on two or three occasions through the giving institutions’ registrar or correspondence offices. From there, where the number of teachers per department and the total teacher number on campus were known, we compared the data with the ratio of respondents from the given institutions and faculties. Following this, we sent the letter of request only to the places where the level of teachers was less than 10%. Exempt from this are the non-Transcarpathian Ukrainian institutions, which are more difficult to reach due to the current conflict, their operation being in many cases challenging and unsure. Likewise, the number of teachers and the nature of their work are hard to know, since there are some who have fled their homes to save their lives (mainly to Transcarpathia), some even leaving the country to do so. Thus, the Hungarian sample is representative per faculty, while the sub-samples from other countries are mainly representative of Hungarian minorities and the majority teachers working with them, mostly in the same institution. In total, 853 filled out the survey and following data cleansing, that number dwindled to 821. We gave the database the name, Central and Eastern European Teachers in Higher Education (CEETHE 2023).

### Participants

The average age of the respondents was 46 (SD = 10.71), and the average amount of years worked in higher education was 17 (SD = 11) years. All the important demographic ad work-related characteristics of the sample is summarized in Table 1.

**Table 1:** Demographic background and work-related characteristics of the sample. Source: CEETHE 2023

Variable	Value	Percentage	*N*
Country	Hungary	62.8	514
	Ukraine	14.1	115
	Romania	13.8	113
	Serbia	4.9	40
	Slovakia	4.4	30
Gender	male	43.7	351
	female	56.3	452
Age	< 29	5.4	43
	30–39	24.2	193
	40–49	35.2	280
	50 − 19	23.2	185
	60–69	10.3	82
	> 70	1.6	13
Academic degree	I do not have	21.1	173
	PhD/DLA	44.6	365
	Candidate of Science (CSc)	5.7	47
	habilitated doctor	17.1	140
	Doctor of Science (DSc)	11.4	93
Discipline	humanities and arts	32.4	264
	STEM and agriculture	23.1	188
	medicine. health and sport	27.9	227
	society and economy	16.7	136
Position	Senior lecturer or lower	58.8	483
	Associate professor or higher	41.2	338
Leader position	No	69.3	569
	yes	30.7	252

### Measurements and variables

The measurements used in the study’s questionnaire were all translated from English. After finalizing the questionnaires, we conducted pilot surveys in each language to verify their validity. The reliability of the applied measurement tools was tested in all languages. Cronbach’s alpha values proved to be adequate, allowing us to proceed with the creation of the complete database.

Based on Kinman et al.’s work [27], we checked the workplace stress- and resources, as well as its perceptions. Using factor analysis (Principal axis factoring method, Varimax rotation), three job stress sources and one resource were identified^1^: *workload*,* performance evaluation*, and the *diversity of teacher roles* as stress sources, as well as *management support* as a resource. Beside this, further factors influencing work performance and wellbeing were inspected, which also could serve as stress- and resources. Since our study’s goal fundamentally was to expose the hardships connected to work, we examined the dimension of *work interference with personal life* from the Work-Life Balance Scale (WLBS) according to Hayman [20], when work disturbs private life.

From among the factors influencing wellbeing, we peered into the realm of Maslach’s Burnout Inventory regarding *emotional exhaustion at work*, and into the *work engagement* using Utrecht Work Engagement-9 scales (UWE-9) [19]. In order to measure *wellbeing*, the Warr’s [43] Affective Wellbeing Scale was used; we asked teachers, in the last few weeks, how often they felt various positive and negative emotions. Therefore, the scales utilized different values (1–5, 1–7) to standardize the values, the factor and components scores were recoded into a 0-100 degree scale, in which 0 represented if the main component was not at all characteristic of the respondent, and 100 if it was completely typical of the respondent. The statistical values, means, and SD of all factors’ main components are contained in Table 2 to follow.

**Table 2:** The statistical values of the examined factors and components (0-100 points scale, *p* = 0.000). Source: CEETHE 2023

The work factors of teachers	Scale/Source	Chronbach’s α	KMO	Total variance explained	Mean	SD	*N*
Management support (resource)	Job stress sources (16 items, not scale) [27]	0.869	0.856	53.045%	58.25	18.69	778
Workload					64.88	19.21	
Diversity of teacher roles					58.65	18.02	
Performance evaluation					58.53	17.41	
Work-private life conflicts	work interference with personal life from the WLBS (6 items, Hayman [20]	0.948	0.905	79.545%	46.76	27.87	806
Emotional exhaustion at work	Maslach Burnout Inventory (5 items, Han et al., [19]	0.911	0.852	73.894%	45.76	25.38	798
Work engagement	UWE-9 (9 items, Han et al., [19]	0.931	0.900	65.441%	70.49	19.76	788
Wellbeing	Affective Wellbeing Scale (12 items, Warr [43]	0.879	0.908	75.773%	59.75	18.89	797

In our study, to calculate physical activity, the IPAQ scale was employed, which measures in a multifaceted fashion the intensive and moderate physical activities done weekly and measure daily to the minute, while also taking into account time sitting (for rest). In comparing these, the amount of daily physical activity during an average week can be predicted, and the respondents are able to be divided up into low (less than 150 min of moderate or 75 min of intensive physical activity weekly), moderate, and high physical activity groups [11]. Considering this, 30.6% of respondents belong to the high activity group, 32.6% to the moderate, and 36.8% to the low activity group.

The demographic background variables we viewed are age, gender, and country of origin; among the work characteristics those variables are academic degree, workplace position, leader position, academic field of expertise.

Our analyses were done through the SPSS29 program, we used the crosstab analysis, Kruskal-Wallis test, and linear regression, and checked the normality with the Kolmogorov-Smirnov test. In all the regression models, multicollinearity was assessed using correlation values, VIF, and tolerance. The results indicated in all cases that there was no multicollinearity between the explanatory and dependent variables. Goodness of Fit was checked by the values of R^2^, ANOVA, and heteroscedasticity, while the normality of the dependent variables was examined through histograms and Normal P-P Plots.

## Results

### The background of physical activity among teachers

We checked what differences exist in daily physical activity and in the groups formed according to physical activity – age, gender, teacher position, field of expertise, degree of education, and types. The results of the Kruskal-Wallis test revealed that habilitated doctors are the most active regarding daily physical activity (*Mean rank* = 464.42), followed by those without a degree (*Mean rank* = 439.47), those with PhD/DLA and CSc degrees being equally active (*Mean rank* = 387.45 and 388.55), and those with a DSc degree (*Mean rank* = 368.16, Chi-square = 16.798, p = 0.002, N = 818) being least active. Serbian and Slovakian teachers were the most active (*Mean rank* = 473.53 and 469.69), followed by Hungarians (*Mean rank* = 423.51), who were trailed by the Ukrainians and Romanians taking the title of „the least active” (*Mean rank* = 358.85 and 355.47, Chi-square = 18.340, *p* = 0.001, *N* = 818). We received weak connections in the physical activity groups by sex and country of origin (Cramer’s V = 0.112 and 0.155). The overrepresentation of men was high, while in the women’s intermediately active group (36.2 and 36.5%), the ratio of men was much lower (37.9%). The 36.9% of men and 38.1% of women belonged to the inactive group (Chi-square = 12.254, *p* = 0.002, *N* = 803). Among Serbian teachers, teachers with high rates of physical activity were overrepresented (47.5%), while those with moderate activity have a smaller ratio (17.5%). In the Ukrainian teacher group, the high activity persons are far fewer (22.6%), while in Slovakia the low activity teachers are more scarce (16.7%). These ratios characterized the majority of Romanians (44.2%) and the largest ratio of Hungarians (36%). Beyond these, we found no other significant connections with other variables.

Teacher background, workplace stress factors, resources and physical activity, and their connections with wellbeing.

The strongest negative correlation of workplace emotional exhaustion (*r*=−0.656, *p* = 000), of work–private life conflicts (*r*=−0.538***) is with wellbeing, while there is a positive relationship with management support (*r* = 0.538***) and work engagement (*r* = 0.560***). Though weak, there is a significant tie between wellbeing, workload (*r*=−0.103***), diversity of teacher roles (*r*=−0.385***) and performance evaluation (*r*=−0.147 ***). Romanian and Serbian teachers have the highest level of wellbeing (*Mean rank* = 457.08 and 403.88), while the Slovakians have the lowest (*Mean rank* = 349.33). Those who have a higher academic degree are characterized by higher wellbeing (*Mean rank* = 457.98 and 422.41), followed by those with PhDs, and the lowest wellbeing attributed to those with a CSc degree (*Mean rank* = 389.37 and 343.50). Those in associate professor positions have higher levels of wellbeing than adjuncts or than someone of a lower position (*Mean rank* = 422.88 and 382.13). We could not find any significant connection to other demographic and workplace factors, or teacher characteristics (Table 3).

**Table 3:** Wellbeing and teachers’ differences of demographic and work characteristic natures in wellbeing (Mean Rank). Source: CEETHE 2023

Background variable		Mean rank	Chi-square	*p*	*N*
Country	Hungary	390.45	10.248	0.036	794
	Ukraine	381.63			
	Romania	457.08			
	Serbia	403.88			
	Slovakia	349.33			
Academic degree	none	376.56	12.492	0.014	794
	PhD/DLA	389.37			
	CSc	343.50			
	Habilitated doctor	422.41			
	DSc	457.98			
Position	Adjunct or lower	382.13	6.060	0.014	797
	Associate professor or higher	422.88			

We tested to see what sort of differences we could find among the physical activity groups; in the stress factors and resources of work, in the work–private life conflicts, in emotional exhaustion, in work engagement, and in wellbeing. We found significant incongruences in four components. The teachers with low activity reached the highest values (*Mean rank* = 415.97, 459.48, and 447.02) in negative stress factors – like diversity of teacher roles –, work–private life conflicts, and emotional exhaustion equally, while the most active teachers achieved the lowest numbers. Regarding wellbeing, we received flipped connections: the group with high physical activity received the highest level of wellbeing (*Mean rank* = 433.87), followed by the intermediately active, and lastly by the inactive group (*Mean rank* = 406.53, and 363.90) (Table 4).

**Table 4:** The differences of teacher groups according to physical activity – workplace stress factors, difficulties, exhaustion, wellbeing (mean rank). Source: CEETHE 2023

	Low	Moderate	High	Chi-square	*p*	*N*
Diversity of teacher roles	415.97	377.15	370.74	6.410	0.041	778
Work-private life conflicts	459.48	383.51	357.29	28.821	0.000	806
Emotional exhaustion at work	447.02	381.75	362.27	20.372	0.000	798
Wellbeing	363.90	406.53	433.87	12.710	0.002	797

**Fig. 1:**
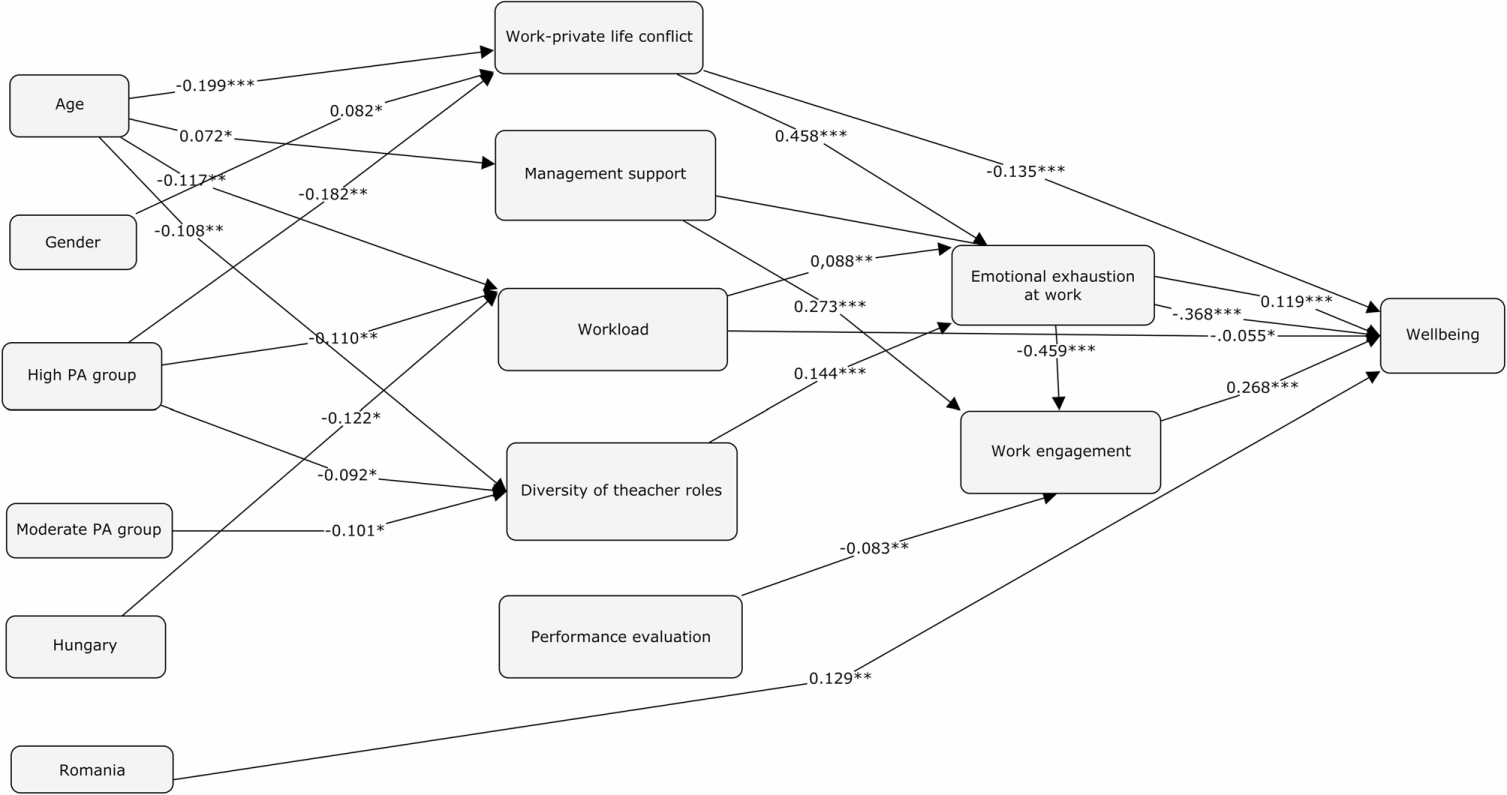
Predictors influencing wellbeing. Source: own editing. Note. Reference values in the case of bivalent variables: men refers to gender; low PA refers to High PA and Moderate PA the teacher’s group; Serbian and Slovakian teachers refer to Hungarian, Romanian and Ukrainian teachers

To investigate the factors influencing wellbeing, we built a model in which we tested the roles of workplace stress factors and resources, work–private life conflicts, emotional exhaustion, and engagement to work in wellbeing, as predictors of belonging to high and moderate physical activity teacher groups, controlling with demographic variables (Fig. 1). One of the most important questions of our research to find the relationship between PA both wellbeing and workplace stress sources and resources, so we examined the role of PA in the case of wellbeing as well as the roles of stress sources and resources. Our question is, whether or not PA contributes to wellbeing, engagement and workplace resources and, if so, how. Our question also explores whether or not it reduces the level of exhaustion and stress sources in work, controlling with demographic and workplace characteristic variables. The model proved to be significant (F(14.673) = 61.829, *p* < 0.001, R^2^ = 0.563), the correlation between explanatory variables in every case being smaller than 0.7, the Tolerance value of the included variables between 0.357 and 0.953, and the VIF value fluttering between 1.050 and 2.800. Six factors have heavy influence on wellbeing. From strongest to weakest they are as follows: emotional exhaustion (β=−0.368, *p* < 0.001, 95%CI[−0.333;−0.219]), engagement in work (β = 0.268, *p* = 0.000, 95%CI[0.199;0.317]), workload (β=−0.055, *p* = 0.039, 95%CI[−0.105;−0.003]), conflicts between work and private life (β=−0.135, *p* < 0.001, 95%CI[−0.143;−0.041]), institutional management support (β=-=0.119, *p* < 0.001, 95%CI[0.063;0.177], and teacher employment in a Romanian institution (β = 0.129, *p* < 0.001, 95%CI[3.103;11.038].

As concerns the predictors as well, we examined the connections, checked the correlation values regarding these models, their multi-collinearity indicators, and the models’ significance, getting back appropriate statistical values. Pertaining to emotional exhaustion at work, we identified work-private life conflicts (β = 0.468, *p* < 0.001, 95%CI[0.357;0.474]), workload (β = 0.088, *p* = 0.001, 95%CI[0.049;0.181]), and diversity of teacher roles (β = 0.144, *p* < 0.001, 95%CI[0.117;0.281] as risk factors. Engagement in work is positively affected by institutional management support as a resource (β = 0.273, *p* < 0.001, 95%CI[0.218;0.357), meanwhile it is decreased by emotional exhaustion at work (β=−0.459, *p* < 0.001, 95%CI[−0.426;−0.290]), and performance evaluation (β=−0.083, *p* = 0.010, 95%CI[−0.168;−0.023]). The perception of institutional management support increases with age (β = 0.072, *p* = 0.050, 95%CI[0.000;0.253]), still the workload decreases (β=−0.117, *p* = 0.001, 95%CI[−0.339;−0.082]). The factors still protecting workload are belonging to a high physical activity group (β=−0.110, *p* = 0.007, 95%CI[−7.897;−1.246]) and working in a Hungarian institution (β=−0.122, *p* = 0.042, 95%CI[−9.415;−0.165]). Diversity of teacher roles as a stress factor is also negatively associated with age (β=−0.108, *p* = 0.003, 95%CI[−0.305;−0.062]), whereas, it also functions as a protective factor, if a teacher has a high (β=−0.092, *p* = 0.025, 95%CI[−6.749;−0.465]) or moderate (β=−0.101, *p* = 0.013, 95%CI[−6.960;−0.811]) level of activity.

The stress factor of performance evaluation has a positive influence if the amount of respondents grows (β = 0.115, *p* = 0.002, 95%CI[1.404;6.528]), but a negative effect if a teacher works in a Hungarian institute (β=−0.135, *p* = 0.025, 95%CI[−8.983;−0.617]). Work-private life conflicts with age characterize respondents less and less, thus it can be considered a negative predictor (β=−0.199, *p* < 0.001, 95%CI[−0.694;−0.339], just like belonging to the moderate (β=−0.147, *p* = 0.000, 95%CI[−13.281;−4.182]) or high physical activity groups (β=−0.182, *p* < 0.001, 95%CI[−15.702;−6.369]). And yet, if a teacher is female, positive relationship can be seen (β = 0.082, *p* = 0.023, 95%CI[0.644;8.599]. The connections can be observed in Fig. 1. below.

## Discussion

In our study, we examined the physical activity background of teachers from five Central and Eastern European countries employed in higher educational institutions (Hungary, Ukraine, Romania, Slovakia, and Serbia). Moreover, we checked its role in workplace stress sources and resources, as well as the total sum of all the factor connections to university teacher wellbeing. At the same time, in accordance with earlier studies [23, 28], the vast majority of subjects belongs to the inactive group (36,8%). In our first hypothesis we assumed younger, male teachers without an academic degree and inhabiting a lower position will be more active physically. The hypothesis has been partially confirmed. We found significant differences in only two main areas in the physical activity groups: among Serbians there were more high physical activity teachers, while in the Ukrainian sample there were far fewer high physical activity teachers. The majority of Romanian respondents are characterized by low activity. In harmony with the results of Saraiva et al. [42], male teachers are more likely to have more physical activity.

As far as workplace stress sources, resources, and wellbeing are concerned, our research results are in one accord with the previous ones, which proved that with increased physical activity, wellbeing’s level rises and stress’ level falls. These results confirm our second hypothesis partly. One of the most vital results of our study was that neither the medium, nor the high-level physical activity had direct connection with burnout and engagement in work in our model. In accordance with stress-buffering hypothesis [9], our results confirm that moderate and high physical activity directly play a preventive role not in burnout itself, but in overcoming the stress sources that lead to burnout. Moderate PA reduces stress caused by diversity of teacher roles, as well as high PA, which is also reduces stress originate from work-private life conflicts and high workload, which are in strong relationship with exhaustion, work engagement and wellbeing.

Our results also show that regardless of age, gender, country of origin, moderate and high physical activity helps decrease the stress that comes with diversity of teacher roles and that they feel the surplus load (it is good to perform well in every area) that accompanies this as less of a hardship. We can assume that doing sports, physical exercise or movement also is counted among a person’s needs, for which time and energy must be set aside. Thus, each day must be planned so that weekly 150 min of moderate exercise or 75 min of intense workout fits in, all the while leaving enough time for completing work-related assignments, and turning attention to private life as well, of which the family is first priority. We can interpret the connections in reverse as well: lower workload, less role overhead can also add to higher physical activity.

Our results regarding exhaustion are in agreement with Moueleu Ngalagou et al.’s [33] results, which seek to draw attention to the strong relationship between an inactive lifestyle and burn-out.

Our last hypothesis was that workplace stressors and exhaustion in work decrease, while PA, workplace resources and work engagement increase the level of teachers’ wellbeing. It was confirmed partly by the results: however, the wellbeing increases with the level of PA, in the multivariable analyzes there was no direct relationship detected between the two. Emotional exhaustion at work, work-life conflicts and high workload are considered as negative predictors, while engagement in work and the management support as positive predictors of wellbeing. The stress of performance evaluation and diversity in teacher role plays also role in wellbeing indirectly. These results confirmed the JD-R theory, and were in harmony with the results of Han et al. [19, 28]. Alqarni [2] also had similar results in his study on workload.

Therein, institutional management support meant that employee work is supported, evaluated, and appreciated by the institutional management. Our results also shed light on the fact that this truly does contribute to work engagement and wellbeing. It is for this very reason that such institutional policy, where teachers receive a certain level of authority to speak into a university’s operation would be critical to implement [3, 29]. Work-private life conflicts, however, increase emotional exhaustion, lessening wellbeing, as Kinman [28] and Fetherstone et al. (2021) both showed in their research. If a teacher is irritated, agitated, feeling overloaded due to his out-of-control work, wellbeing will be lowered, and he/she will experience imbalance and more conflict in both his personal and work life [5].

What is more, via work engagement and emotional exhaustion, other workplace sources of stress and resources affect wellbeing. Stress caused performance evaluation decreases, management support as a resource grows work engagement [30]. The results of the hypothesis testing are summarized in the table below (Table 5).

**Table 5:** Result of hypothesis testing

Hypothesis	Results	Result of hypothesis testing
H1. Younger male teachers without an academic degree and lower position will be more active physically.	We found significant differences in only two main areas in the physical activity groups: country and gender.	partially confirmed
H2. The level of workplace stress sources and emotional exhaustion decreases, while perception of workplace resources, level of engagement increase with the level of PA.	PA had no direct connection with burnout and engagement in work but directly played a preventive role in overcoming the stress sources that lead to burnout and reduce work engagement.	partially confirmed
H3. Workplace stressors and exhaustion in work decrease, while PA, workplace resources and work engagement increase the level of teachers’ wellbeing.	PA had no direct relationship with wellbeing. Emotional exhaustion at work, work-life conflicts and high workload are considered as negative predictors, while engagement in work and the management support as positive predictors of wellbeing	partially confirmed

The demographic factors, however, do not influence wellbeing. Wellbeing is highest among Romanians, just as it is among those in high positions and those who have high academic degrees. The higher level of wellbeing among Romanian teachers can be explained by the generally higher level of subjective wellbeing in the country: according to the World Happiness Report 2023, Romania was ranked 24th on the basis of a three-year average, by far the best place among the countries investigated and also among other CEE countries, followed only by the Czech Republic [21]. The selection effect could also play a role, given the rather small Romanian sample, and perhaps only more motivated (and thus happier) teachers contributed.

Although the Ukrainian respondents did not score the lowest in well-being, and working at a Ukrainian higher education institution showed no association with well-being, workplace stress sources or resources, there is no doubt that the ongoing war serves as a constant source of anxiety in their daily lives. Moreover, this ever-present threat affects not only them but also the neighbouring countries examined in our study, which have taken in the most war refugees. The reason why this continuous hardship did not manifest in the results of the Ukrainian respondents can be attributed to the focus of our research, we specifically examined work-related difficulties, challenges, and resources. In these areas, the Ukrainian respondents performed well. Compared to teachers from other countries, they rated their working conditions, opportunities for professional development, and social support the highest, all of which are crucial resources in their work [30].

Thus, while they face daily struggles caused by the war—such as the separation of family and friends, the constant fear of male family members being conscripted, power outages, and rising costs, etc.—their workplace provides a safe environment that offers opportunities for promotion, development, and social support. Additionally, it serves as a distraction from the ongoing war-related tensions. Our interview-based research also highlighted that war-related difficulties were primarily mentioned in the context of life outside the university, such as limited entertainment, recreation, and leisure opportunities [29].

This may be because, in the border region of Ukraine examined, institutions have adapted to the war-induced circumstances to ensure that education continues as smoothly as possible including establishing shelters, acquiring generators, transitioning to online or hybrid teaching, and ensuring that education is also available in the state language at minority Hungarian institutions.

Our study broadly examined the work characteristics, hardships and strengths, and connections of Central and Eastern European teachers, but it has several limitations. The literature identifies job satisfaction as an important workplace outcome that can be both a performance indicator and a predictor. We did not, however, investigate this in our research. In our questionnaire we asked about the level of respondent satisfaction with academic performance. Investigating the background to this, as well as other important influencing factors such as job insecurity, working hours could result in further directions for research.

In our research, we primarily focused on the role of workplace factors and physical activity in relation to well-being. We did not investigate, among other things, the effects of war as the most significant source of stress in Ukraine, which profoundly impacts the daily lives, well-being, and mental health of teachers living there. Therefore, the relationships we identified and our conclusions are only of limited validity for this group. Although we aimed for a representative sample in most institutions, we encountered obstacles in collecting the data in several institutions and countries, confining the validity of our results and preventing them from being generally applied to the institutions and, in particular, the countries studied. However, as pointed out by Mudrak et al. [35], there are few studies on the work and well-being of academics in the CEE region, so our findings have incomplete relevance to this topic and region.

Thus, our results do contribute to the academic discourse on the topic and to the preparation of education policy decisions.

Based on our findings, we recommend implementing institutional prevention programs at the examined higher education institutions. These programs would contribute to curtail workplace burnout, help teachers cope with stress, and increase their engagement. To this end, it is crucial that they have the support of institutional leaders, that they feel their work is valued, particularly by department heads. The evaluation of performance must be clear, transparent and equally applicable to everyone, with teachers involved in its development. To cope with stress effectively, mental health services focusing on teaching methods for dealing with work-life conflicts and workloads are irreplaceable.

Along with the former, according to our findings, regular physical activity is also an effective tool. Based on this, we recommend providing sports programs for teachers, free of charge, offering regular opportunities to exercise together as a community, so they can motivate each other and develop interpersonal relationships. The University of Debrecen’s Employment Exercise Program aims to achieve this by using a questionnaire to record all sports activities undertaken by employees of the university’s various units (faculties and institutes), which are then converted into points. These units compete with each other every month, and the unit that scores the most points is, at a ceremony, awarded the title of “the most athletic unit of the month”. This motivates individual employees to exercise not only for themselves, but also for their unit. The competition encourages members of each unit to exercise more and spend more time together doing sports. This playful competition seeks to foster a strong sense of community among participants at the university who are committed to exercise.

## Conclusion

Modern lifestyles present many challenges to individuals, especially those in higher education. This phenomenon can be particularly critical for university teachers, who must cope with an intense pace of work while achieving excellence in teaching and research. Subjective wellbeing is also a frequent concern among university teachers, as the negative effects of work-related stress “spill over” into the home environment, limiting opportunities for rest and recovery, leading to a decline in health and performance [1].

Our results shed light on physical activity’s role in decreasing workplace stress sources, thus this positive connection can be used in coming up with a program to popularize physical activity, which is specifically for teachers. It would be motivating for teachers if these programs would, with the support of the leadership, take place during work hours. Besides this, the provision of time management programs for teachers would aid them in doing their jobs in more a organized manner and with a more fruitful use of their time, by finding the balance between work and private life.

Furthermore, if the university would like to improve its employees’ wellbeing, then it would be equally essential to emphasize institutional management support, which would provide clear-cut expectations and tasks, transparent institutional strategies, goals, and plans. This would mean that the leadership should recognized and trust teachers, involving them in the life and operation of the university.

## Data Availability

The data that support the findings of this study are available from the corresponding authors, but restrictions apply to their availability. These were used under license for the current study, and, so, are not accessible by the public. Data are however available from the corresponding authors upon reasonable request and with permission of the School Ethics Committee of Doctoral Program on Educational Sciences at the University of Debrecen.

## Footnotes

1 The factors were created by the authors; the original measurement was not divided into factors or subscales. The author did not assign a name to the items of job stressors as a scale; the items were utilized from the study.
